# Early versus delayed administration of norepinephrine in patients with septic shock

**DOI:** 10.1186/s13054-014-0532-y

**Published:** 2014-10-03

**Authors:** Xiaowu Bai, Wenkui Yu, Wu Ji, Zhiliang Lin, Shanjun Tan, Kaipeng Duan, Yi Dong, Lin Xu, Ning Li

**Affiliations:** Research Institute of General Surgery, Jinling Hospital, Nanjing Clinical School of Second Military Medical University, No.305, Zhongshan East Road, Nanjing, P.R. China

## Abstract

**Introduction:**

This study investigated the incidence of delayed norepinephrine administration following the onset of septic shock and its effect on hospital mortality.

**Methods:**

We conducted a retrospective cohort study using data from 213 adult septic shock patients treated at two general surgical intensive care units of a tertiary care hospital over a two year period. The primary outcome was 28-day mortality.

**Results:**

The 28-day mortality was 37.6% overall. Among the 213 patients, a strong relationship between delayed initial norepinephrine administration and 28-day mortality was noted. The average time to initial norepinephrine administration was 3.1 ± 2.5 hours. Every 1-hour delay in norepinephrine initiation during the first 6 hours after septic shock onset was associated with a 5.3% increase in mortality. Twenty-eight day mortality rates were significantly higher when norepinephrine administration was started more than or equal to 2 hours after septic shock onset (Late-NE) compared to less than 2 hours (Early-NE). Mean arterial pressures at 1, 2, 4, and 6 hours after septic shock onset were significantly higher and serum lactate levels at 2, 4, 6, and 8 hours were significantly lower in the Early-NE than the Late-NE group. The duration of hypotension and norepinephrine administration was significantly shorter and the quantity of norepinephrine administered in a 24-hour period was significantly less for the Early-NE group compared to the Late-NE group. The time to initial antimicrobial treatment was not significantly different between the Early-NE and Late-NE groups.

**Conclusion:**

Our results show that early administration of norepinephrine in septic shock patients is associated with an increased survival rate.

## Introduction

Septic shock is the most challenging problem in critical care medicine and has a very high mortality [1-3]. Owing to the complex pathophysiology, the outcomes for septic shock patients remain disappointing. Rivers *et al*. demonstrated that early goal-directed therapy provided significant benefits with respect to outcomes for septic shock patients [4]. However, massive fluid resuscitation may increase extravascular edema, aggravate pulmonary dysfunction and compromise tissue oxygenation [5,6]. Therefore, investigating the rational use of vasopressors in septic shock is very important. Thus far, most studies have focused on the rational use of different types of vasopressors [7-9], and the third edition of the guidelines for management of severe sepsis and septic shock also concentrates on the choice of vasopressors [10]. However, it is the timing of vasopressor therapy, rather than the specific agent, that appears to be crucial [11]. The current guidelines recommend that vasopressors (norepinephrine as the first choice) be administered for hypotension refractory to initial fluid resuscitation and to maintain a mean arterial pressure (MAP) ≥65 mm Hg, and this should be completed within six hours [10]. According to the guidelines, physicians currently prefer fluid resuscitation without vasopressors until a lack of hypotension correction is confirmed. However, this may result in prolonged hypotension, and valuable time may have passed. For example, some vital organs could be damaged irreversibly because of low perfusion. In addition, some researchers have argued that early initiation of norepinephrine administration, especially when hypovolemia has not been resolved, can adversely affect vital organ microcirculatory blood flow and perfusion [12,13]. Hence, this retrospective study examined the relationship between delay in initial norepinephrine administration and hospital mortality and investigated the effects of early norepinephrine administration on septic shock.

## Material and methods

This retrospective cohort study was conducted in two general surgical intensive care units (ICUs) of a tertiary care hospital, Jinling Hospital in Nanjing, China, which mainly admit patients with severe complications after surgery and severe trauma. In these ICUs, vasopressors were administered according to the Surviving Sepsis Campaign (SSC) guidelines [14]. First, initial fluids resuscitation termed early goal-directed therapy and early antimicrobial treatment were administered as soon as sepsis-induced tissue hypoperfusion was recognized [4]. Then, when the physicians judged that hypotension did not respond to initial fluids resuscitation, vasopressor therapy was initiated with the aim of maintaining MAP at least 65 mm Hg and norepinephrine was the first-choice vasopressor.

The study was approved by the Institutional Review Board of Jinling Hospital. Because all data were anonymous and collected retrospectively, the Institutional Review Board of Jinling Hospital waived the need for informed consent.

### Study patients

**Figure 1:**
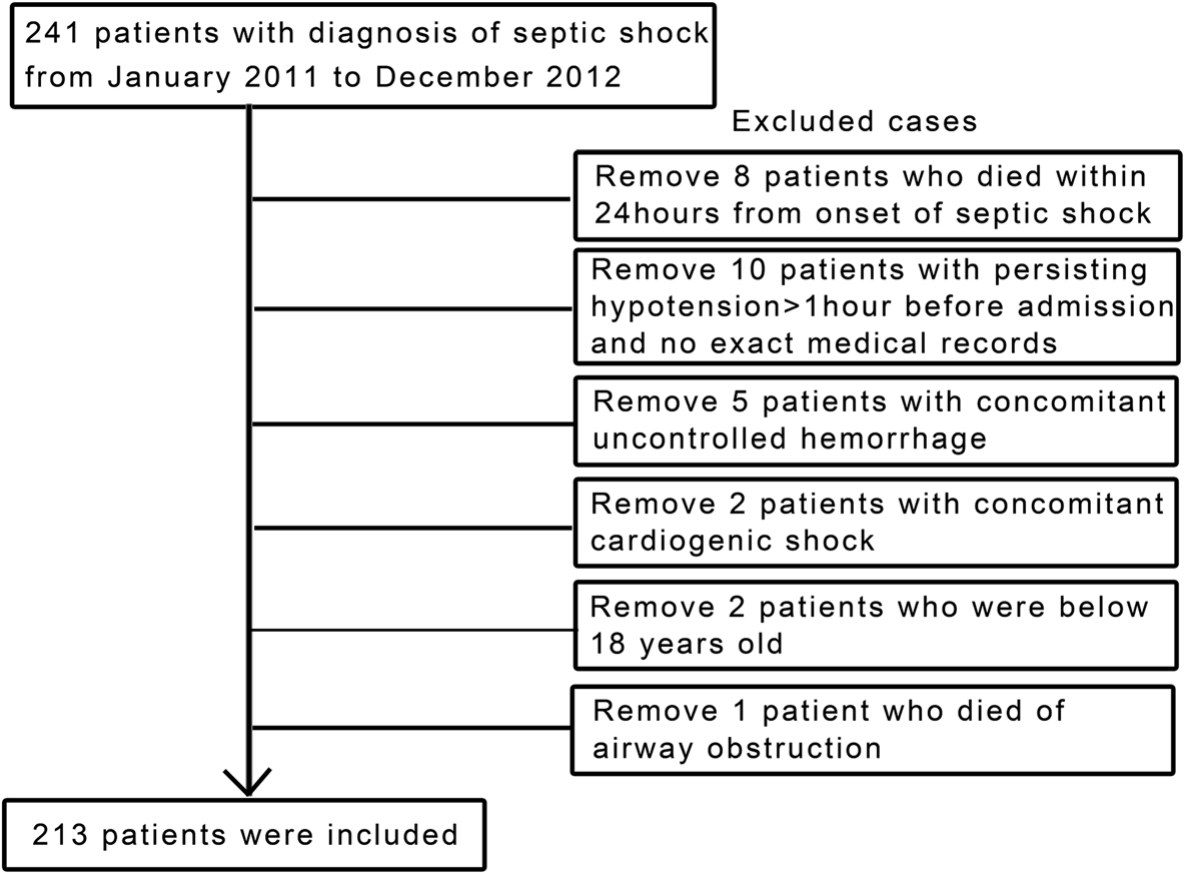
**Inclusion and exclusion flowchart.**

We reviewed the medical records of all adult (≥18 years of age) patients with a diagnosis of septic shock as described by the SCCM/ESICM/ACCP/ATS/SIS International Sepsis Definitions Conference [15]. Sepsis was defined as the presence of infection (documented or probable) with systemic manifestations of infection [15]. Septic shock was defined as sepsis-induced, persisting hypotension, which was defined as systolic blood pressure <90 mm Hg, a decrease of 40 mm Hg in systolic pressure from the patient’s baseline or MAP <65 mm Hg. An episode of hypotension was deemed to represent septic shock when (1) hypotension persisted from the onset despite adequate fluid resuscitation (30 mL/kg crystalloid fluids) or (2) hypotension was only transiently improved (for <1 hour) with adequate fluid resuscitation. All included patients were treated from January 2011 to December 2012 and had no other obvious cause of shock. The inclusion flowchart is described in Figure 1.

### Data collection

The collected data included age, gender, body weight, serum lactate, MAP, the Acute Physiology and Chronic Health Evaluation (APACHE) II scores based on the worst values obtained within 24 hours after the onset of septic shock, documented infections, microbiologic results, primary infection site, volume (L) of intravenous fluids administered within 6 and 24 hours after the onset of septic shock (including crystalloid, colloid and blood products), quantity (mg) of norepinephrine administration within the 24 hours after the onset of septic shock, time (hours) to initial norepinephrine administration, time (hours) to initial antimicrobial treatment, effective antimicrobial therapy, corticosteroid treatment, norepinephrine duration, hypotension duration, ICU duration and 28-day mortality. Two investigators independently reviewed the included records using a standardized data collection form. A third author resolved any discrepancies.

### Definitions

We calculated an APACHE II score to assess the severity of illness. Time to initial administration of norepinephrine was calculated from the onset of septic shock to initial norepinephrine administration. Effective antimicrobial therapy was declared when the first administration of a new antimicrobial to which the pathogen was susceptible or that matched national guidelines in cases of culture-negative septic shock was initiated within six hours after the onset of septic shock. Time to initial antimicrobial treatment was calculated from the onset of septic shock. Corticosteroid treatment was defined to have occurred if it was started within six hours after the onset of septic shock. Documented infection was concluded when a plausible pathogen from the blood or infection site was identified with a compatible syndrome or when infection was supported by a definitive surgical, radiologic or pathologic diagnosis. All other infections were declared to be suspected. The primary infection site was ascertained by the consensus of three researchers. ICU duration was calculated from the time of patient admission instead of the onset of septic shock.

### Statistical analysis

We compared the Early-NE group to the Late-NE group (which received norepinephrine <2 hours and ≥2 hours after the onset of septic shock, respectively) based on the odds ratio (OR) analysis. Data were analyzed with SPSS software, version 19.0 (SPSS, Inc., Chicago, IL, USA). Values were expressed as the means ± standard deviation (SD) (continuous variables) or as a percentage of the group from which they were derived (categorical variables). Continuous variables were compared using the Student t-test for normally distributed variables and the Mann–Whitney U test for non-normally distributed variables. Categorical variables were compared using the chi-square or Fisher’s exact test. All comparisons were unpaired, and all *P* values were two-tailed. A *P* value ≤0.05 was considered to indicate statistical significance.

Logistic regression analysis was performed to examine 28-day mortality as a function of time to initial norepinephrine administration. The primary outcome variable was 28-day mortality, and the primary independent variable was time to initial norepinephrine administration as a continuous variable. In addition, multivariate logistic regression was undertaken to examine the independent effect of variables on 28-day mortality, and the results are reported as adjusted ORs of death with their corresponding 95% confidence intervals (CI).

## Results

These analyses included 213 patients. All patients received norepinephrine as the initial vasopressor agent. The overall 28-day mortality was 37.6%.

Initial serum lactate level was 4.7 ± 1.5 mmol/L and the APACHE II score was 28.4 ± 4.2. Infections were documented in 191 cases (89.7%); the remaining cases represented suspected infections without a positive culture or definitive surgical, radiologic, biopsy, or autopsy evidence of infection. Positive cultures were identified in 175 cases (82.2%). A plausible microbial pathogen was isolated from the blood in 132 cases (62.0%).

**Figure 2:**
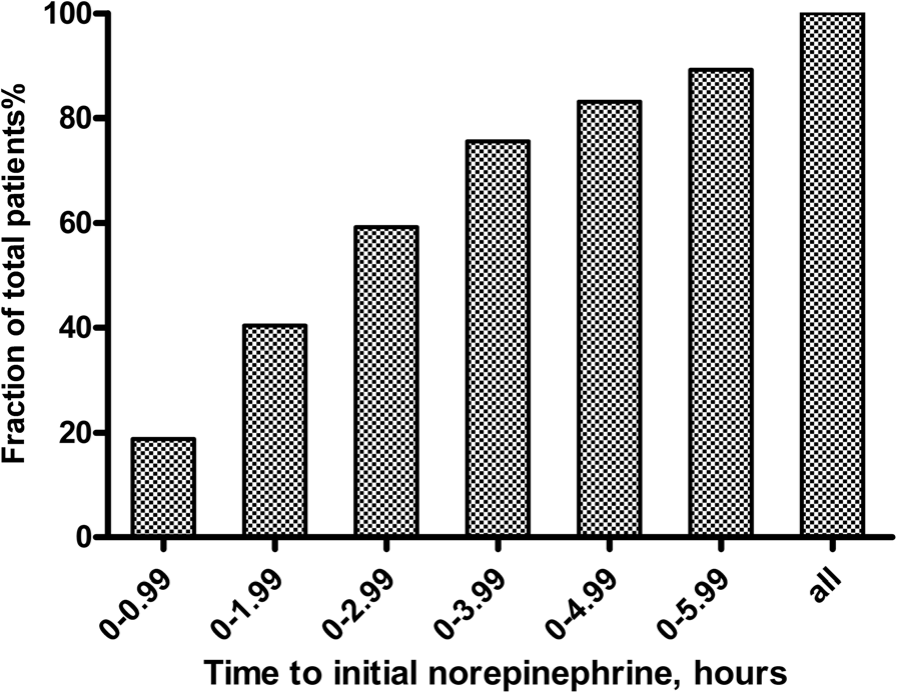
**Cumulative norepinephrine initiation.** Bars represent the fraction of patients who received initial norepinephrine administration from the time of septic shock onset to the indicated ending time point.

**Figure 3:**
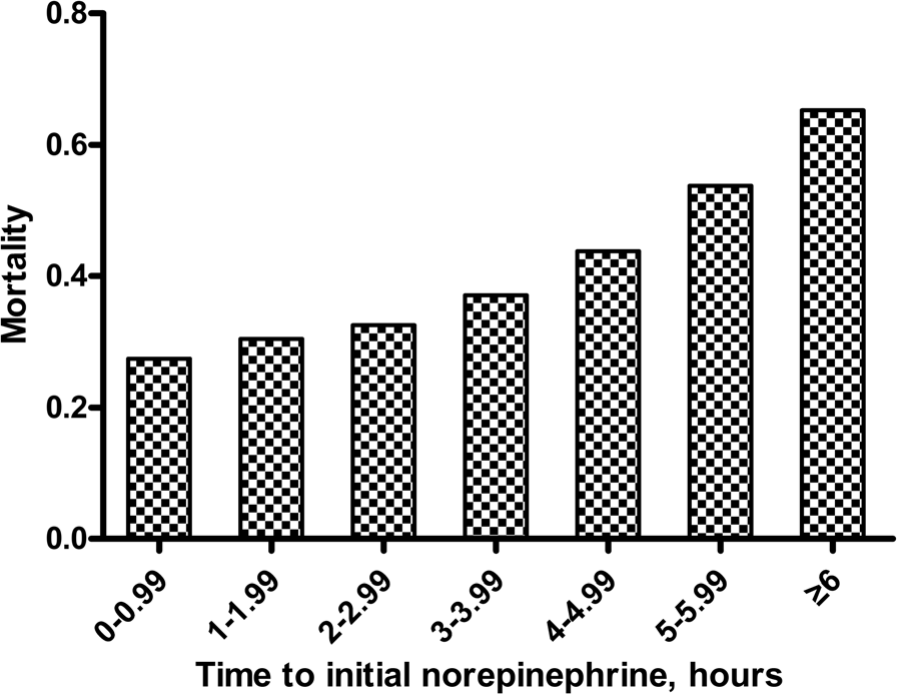
**Mortality of patients whose initial norepinephrine administrations were within the indicated time interval.**

**Figure 4:**
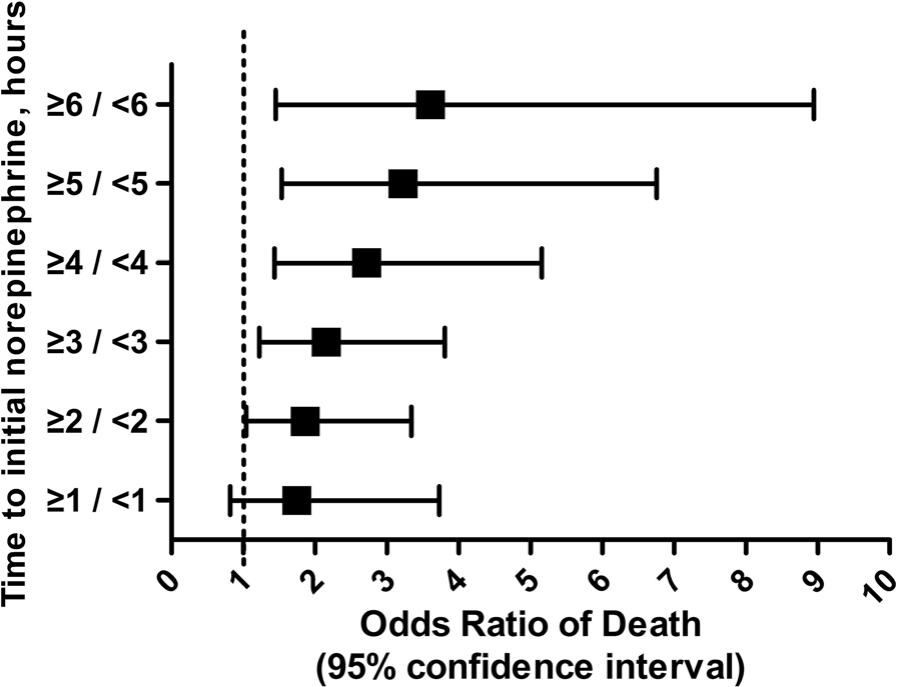
**Relationship between different norepinephrine administration delays and hospital mortality expressed as odds ratio of death.** Bars represent 95% confidence interval. The y-axis represents different norepinephrine administration time delays from the onset of septic shock.

There were no significant differences between survivors and non-survivors with respect to age, gender or the frequencies of documented infections and positive cultures or source of infection. Initial serum lactate levels and APACHE II scores were significantly higher in the non-survivors than in the survivors (Table 1). Survivors received more intravenous fluids within 6 hours (3.4 ± 0.9 L versus 3.0 ± 0.9 L; *P* =0.003) but less intravenous fluids within 24 hours (6.5 ± 0.8 L versus 6.9 ± 0.5 L; *P* <0.001) than the non-survivors. They also received less norepinephrine within 24 hours (29.4 ± 9.7 mg versus 34.8 ± 9.6 mg; *P* <0.001). Survivors also had a shorter time to initial norepinephrine administration (2.7 ± 2.1 hours versus 3.8 ± 2.9 hours; *P* =0.002), had a shorter time to initial antimicrobial treatment (1.4 ± 1.2 hours versus 2.2 ± 1.8 hours; *P* =0.001) and received effective antimicrobial therapy more frequently (72.9% versus 56.3%; *P* =0.012) (Table 2). The survivors were treated with norepinephrine for a shorter duration (2.4 ± 0.6 days versus 3.4 ± 0.9 days; *P* <0.001; Table 2). The frequency of corticosteroid treatment was similar in the survivors and the non-survivors. Overall, the average time to initial norepinephrine administration was 3.1 ± 2.5 hours. A total of 18.8% of all patients received norepinephrine administration within 1 hour after the onset of septic shock, 40.4% within 2 hours and 9.9% of patients ≥6 hours after the first occurrence of septic shock (Figure 2). The relationship between hospital mortality and the time to initial norepinephrine administration is shown in Figure 3. Mortality was 27.5% if norepinephrine administration was initiated <1 hour after the onset of septic shock, 30.4% if initiated from 1 to 2 hours after the onset and 65.2% if initiated ≥6 hours after the onset. Twenty-eight-day mortality was significantly higher in patients who received norepinephrine ≥2 hours after septic shock onset and increased when compared to those who received it <2 hours after onset (OR for death =1.86; 1.04 to 3.34; *P* =0.035; Figure 4). The OR of death was 2.16 (1.23 to 3.81, *P* =0.007) when norepinephrine was administered after three hours and 3.61 (1.45 to 8.95, *P* =0.004) when given after six hours (Figure 4).

**Table 1:** **Demographic data and baseline characteristics: Phase I**

**Characteristic**	**28-day survivors (n = 133)**	**28-day non-survivors (n = 80)**	***P*** **value**
Age (years)	58.2 ± 11.9	59.5 ± 14.4	0.507
Gender, number (%)			0.684
Male	71 (53.4)	45 (56.2)	
Female	62 (46.6)	35 (43.8)	
Weight (kg)	64.0 ± 13.8	64.7 ± 13.2	0.708
Serum lactate at septic shock onset (mmol/L)	4.3 ± 1.4	5.3 ± 1.5	<0.001
APACHE II score	27.2 ± 3.9	30.4 ± 3.9	<0.001
Documented infection, number (%)	117 (88.0)	74 (92.5)	0.293
Culture positive, number (%)	108 (81.2)	67 (83.8)	0.638
Primary infection site, number (%)			0.675
Respiratory	23 (17.3)	18 (22.5)	
Intra-abdominal	46 (34.6)	28 (35.0)	
Genitourinary	13 (9.8)	5 (6.3)	
Skin and soft tissue	5 (3.8)	2 (2.5)	
Intravascular catheter	15 (11.3)	10 (12.5)	
Bloodstream	8 (6.0)	8 (10.0)	
Other	23 (17.3)	9 (11.3)

**Table 2:** **Therapeutic intervention and secondary outcomes: Phase I**

**Characteristic**	**28-day survivors (n = 133)**	**28-day non-survivors (n = 80)**	***P*** **value**
24-hour norepinephrine administration (mg)	29.4 ± 9.7	34.8 ± 9.6	<0.001
Time to initial norepinephrine administration (h)	2.7 ± 2.1	3.8 ± 2.9	0.002
Time to initial antimicrobial treatment (h)	1.4 ± 1.2	2.2 ± 1.8	0.001
Volume of intravenous fluids within 6 h (L)	3.4 ± 0.9	3.0 ± 0.9	0.003
Volume of intravenous fluids within 24 h (L)	6.5 ± 0.8	6.9 ± 0.5	<0.001
Effective antimicrobial therapy, number (%)	97 (72.9)	45 (56.3)	0.012
Corticosteroid treatment, number (%)	78 (58.6)	50 (62.5)	0.578
Norepinephrine duration (days)	2.4 ± 0.6	3.4 ± 0.9	<0.001
ICU duration (days)	11.2 ± 5.7	10.8 ± 5.4	0.646

In univariate logistic regression analysis, the time from the onset of septic shock to initial norepinephrine administration was an important determinant of 28-day mortality. The OR of death was 1.20 per hour delay (1.07 to 1.35, *P* =0.002), that is, every 1-hour delay was associated with a 20.4% increased probability of death.

The 28-day mortality was 29.1% in the Early-NE group (within two hours) and 43.3% in the Late-NE group (after two hours). There were no significant differences with respect to age, gender, weight, APACHE II score, documented infections, positive cultures and primary infection sites between the Early-NE and the Late-NE group (Table 3). Serum lactate levels at the onset of septic shock were even significantly higher in the Early-NE group than in the Late-NE group (Table 3).

**Table 3:** **Demographic data and baseline characteristics: Phase II**

**Characteristic**	**<2 hours (number = 86)**	**≥2 hours (number = 127)**	***P*** **value**
Age (years)	57.7 ± 12.2	59.4 ± 13.4	0.361
Gender, number (%)			0.963
Male	47 (54.7)	69 (54.3)	
Female	39 (45.3)	58 (45.7)	
Weight (kg)	64.7 ± 14.4	64.0 ± 12.9	0.731
Serum lactate at septic shock onset (mmol/L)	5.0 ± 1.7	4.4 ± 1.4	0.011
APACHE II score	28.6 ± 4.6	28.2 ± 4.7	0.231
Documented infection, number (%)	77 (89.5)	114 (89.8)	0.957
Culture positive, number (%)	73 (84.9)	102(80.3)	0.393
Primary infection site, number (%)			0.960
Respiratory	17 (17.3)	24 (22.5)	
Intra-abdominal	32 (34.6)	42 (35.0)	
Genitourinary	7 (9.8)	11 (6.3)	
Skin and soft tissue	3 (3.8)	4 (2.5)	
Intravascular catheter	10 (11.3)	15 (12.5)	
Bloodstream	7 (6.0)	9 (10.0)	
Other	10 (17.3)	22 (11.3)	

**Figure 5:**
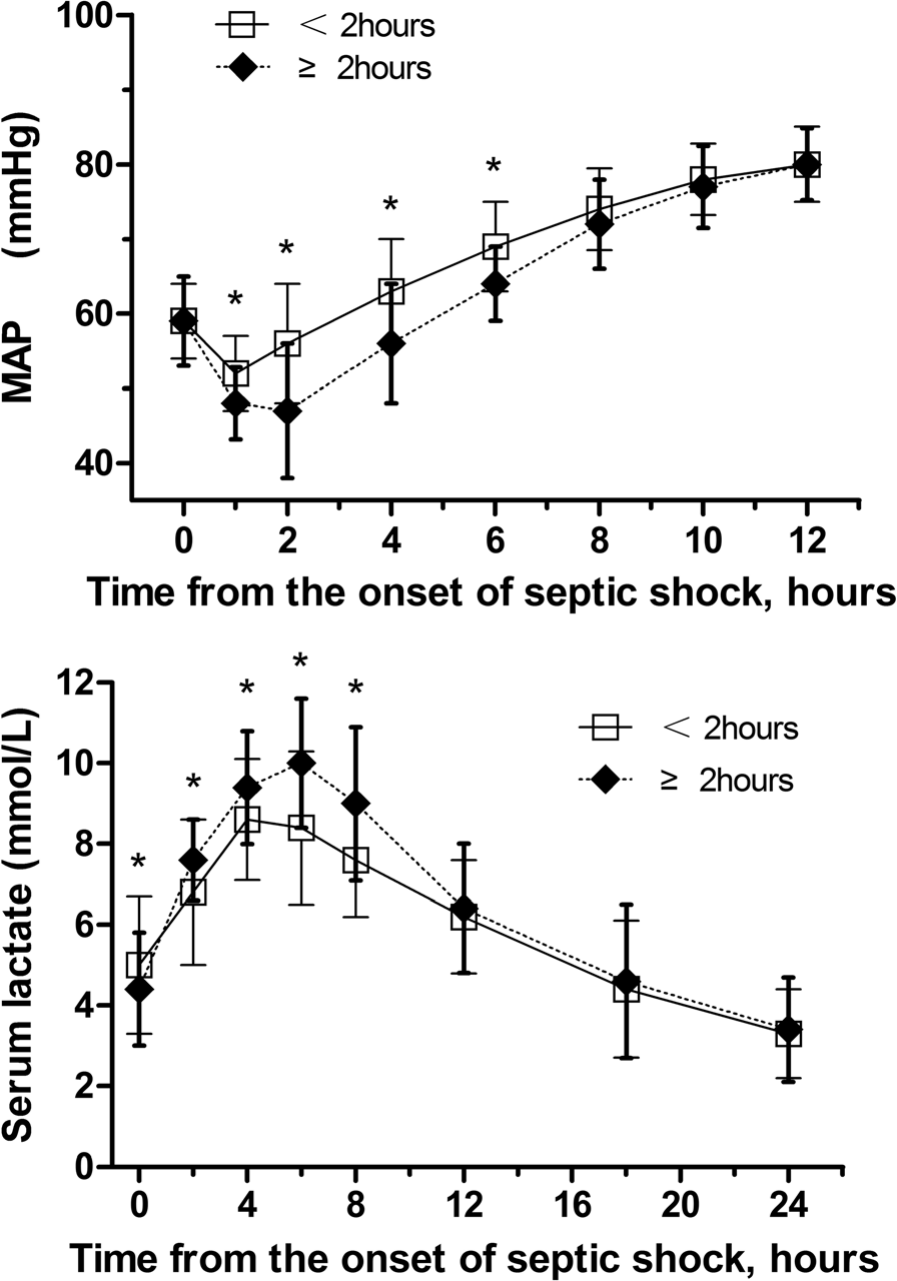
**Changes of MAP and serum lactate level following the onset of septic shock in the Early-NE group and the Late-NE group.** Bars represent standard deviation. *P <0.05 for the comparison of the Early-NE group to the Late-NE group. MAP, mean arterial pressure; NE, norepinephrine.

Patients in the Early-NE group received less intravenous fluid within 24 hours (6.2 ± 0.6 L versus 6.9 ± 0.7 L; *P* <0.001) and less norepinephrine within 24 hours (29.4 ± 9.7 mg versus 32.8 ± 10.0 mg; *P* =0.013) (Table 4). There was no significant difference between the two groups in the onset of intravenous fluid therapy, the time to initial antimicrobial treatment, the frequency of effective antimicrobial therapy and the frequency of corticosteroid treatment (Table 4). The duration of hypotension was shorter in the Early-NE group (4.6 ± 1.2 hours versus 6.1 ± 1.0 hours; *P* <0.001), and the duration of norepinephrine was also statistically shorter in these patients (2.6 ± 0.6 days versus 2.9 ± 1.0 days; *P* =0.001). There was no significant difference in the duration of ICU stay. Mean arterial pressure was higher at one, two, four and six hours after the onset of septic shock and serum lactate levels at two, four, six and eight hours after the onset of septic shock were significantly lower in the Early-NE group than in the Late-NE group (*P* <0.05, Figure 5). Multivariate logistic regression analysis identified the time to initial norepinephrine administration, the time to initial antimicrobial treatment, serum lactate level at the onset of septic shock, APACHE II score, intravenous fluid therapy within six hours and effective antimicrobial therapy as independent determinants of 28-day mortality (Table 5).

**Table 4:** **Therapeutic intervention and secondary outcomes: Phase II**

**Characteristic**	**<2 hours (number = 86)**	**≥2 hours (number = 127)**	***P*** **value**
24-hour norepinephrine administration (mg)	29.4 ± 9.7	32.8 ± 10.0	0.013
Time to initial antimicrobial treatment (h)	1.6 ± 1.4	1.7 ± 1.5	0.126
Volume of intravenous fluids within 6 h (L)	3.1 ± 0.9	3.3 ± 0.8	0.092
Volume of intravenous fluids within 24 h (L)	6.2 ± 0.6	6.9 ± 0.7	<0.001
Effective antimicrobial therapy, number (%)	55 (64.0)	87 (68.5)	0.489
Corticosteroid treatment, number (%)	54 (62.8)	74 (58.3)	0.508
Norepinephrine duration (days)	2.6 ± 0.6	2.9 ± 1.0	0.001
Hypotension duration (h)	4.6 ± 1.2	6.1 ± 1.0	<0.001
ICU duration (days)	10.7 ± 6.0	11.2 ± 5.2	0.520

**Table 5:** **Multivariate logistic regression analysis of independent risk factors for 28-day mortality**

**Variable**	**Adjusted Odds Ratio of Death**	**95% Confidence interval**	***P*** **value**
Risk factors			
Time to initial norepinephrine administration (h)	1.392	1.138–1.702	0.003
Time to initial antimicrobial treatment (h)	1.330	1.067–1.659	0.011
Serum lactate at septic shock onset (mmol/L)	1.710	1.174–2.537	0.005
APACHE II score	1.243	1.096–1.409	<0.001
Protective factors			
Effective antimicrobial therapy	0.477	0.231–0.982	0.040
Volume of intravenous fluids within 6 h (L)	0.676	0.468–0.977	0.033

*Hosmer-Lemeshow goodness-of-fit test*, *P* =*0.627*.

## Discussion

The present data show that the time from the onset of septic shock to initial norepinephrine administration is an important determinant of survival. The risk of death was significantly increased with longer delay in norepinephrine initiation after the onset of septic shock. For every one-hour delay in norepinephrine initiation within six hours after the onset of septic shock, the mortality increased by 5.3%. This could not be attributed to the more severe septic shock in the delayed norepinephrine initiation group. Initial blood pressure levels were similar. On the contrary, patients in the Early-NE group had higher serum lactate levels than those in the Late-NE group.

Early norepinephrine administration reduced the duration and the total dose of norepinephrine treatment and shortened the duration of shock with less intravenous fluid requirements (a 10% decrease in the first 24 hours for treatment). These data suggest that in the case of severe septic shock, early norepinephrine administration should not be delayed. According to the SSC guidelines [10,14], fluids and antimicrobials should be administered as early as possible in patients with septic shock, but the optimal timing of vasopressor administration is less clear. The patients with septic shock receiving vasopressors early or late depend on the patient’s response to initial fluid resuscitation and the judgment of the physicians. It remains difficult to determine if late administration of norepinephrine reflects a poor evaluation of the disease severity of the patient and if the higher mortality is related to a globally less appropriate management of the patients.

Our results also indicate that although the time to initial antimicrobial treatment was not significantly different between the Early-NE group and the Late-NE group, it was significantly longer in the non-survivor group than the survivor group and it is a critical determinant of outcome. In the multivariate logistic regression analyses, time to initial antimicrobial treatment was a significant independent risk factor for 28-day mortality, a result consistent with prior observations [16,17]. Kumar *et al*. reported a linear increase in the risk of mortality for each hour delay in antibiotic therapy [16]. The percentage of patients with positive blood cultures was 62% in our study compared to around 40% in other studies [16]. Some factors may have influenced these results. First, our ICUs were general surgical ICUs and most patients admitted to them had severe intra-abdominal infections or intestinal fistulae, whereas most of the other studies were conducted in medical ICUs and mixed ICUs. The bias between the different patient characteristics might be one reason for the different results. Second, most patients in our study underwent multiple surgical interventions, including surgical puncture, which would increase the opportunity for pathogens to enter the blood. Other factors could have influenced the results of positive blood cultures as well (for example, nosocomial infection).

Additionally, our data show that the time to initial norepinephrine administration, the time to initial antimicrobial treatment, serum lactate level at the onset of septic shock, APACHE II score, intravenous fluids within six hours and effective antimicrobial therapy were independently associated with hospital mortality.

The results of this study are consistent with those reported in some previous studies [18,19]. In a retrospective study, Morimatsu *et al*. reported that survival of septic shock patients treated with early and exclusive norepinephrine administration (median time 1.3 hours) compared favorably with that predicted by severity scores [19]. In rats with septic shock, Sennoun *et al*. compared the effects of early versus delayed norepinephrine administration, and showed that the mesenteric/aortic blood flow ratio was higher and blood lactate concentrations lower in an early compared to a late norepinephrine group [18]. Additionally, early norepinephrine administration spared approximately 30% of the fluid volume required in the late or non-norepinephrine groups [18]. In another retrospective study, Subramanian *et al*. found that liberal vasopressor use was not associated with less progression to organ failure in septic shock [20]. However, the volume of fluids received within six hours was significantly greater in the liberal than in the conservative group and they used different types of vasopressors, including dopamine, phenylephrine, norepinephrine and vasopressin. Their study was also conducted in a medical ICU, so that differences in patient characteristics may have influenced the results as well.

Septic shock is characterized by hypotension, related in part to absolute and relative hypovolemia. The former is the result of vascular leakage caused by endothelial injury and the latter is the result of systemic vasodilation [21]. In addition, sepsis can result in down-regulation of norepinephrine receptors [22]. Increased MAP with norepinephrine may improve organ perfusion and this may result in lower serum lactate levels. Therefore, expedited norepinephrine administration can be a rational approach to rapidly restore organ perfusion. One may, therefore, be recommended to initiate norepinephrine administration simultaneously with fluid resuscitation at the onset of septic shock. Obviously, this strategy should not prevent adequate fluid resuscitation, as one must pay attention to a so-called ‘vasoconstrictor-masked hypovolemia’ [23].

Our study has several important limitations. First, the retrospective trial design limits our ability to identify precisely the causes for delay in initial vasopressor administration and to determine a causal relationship between the delay in norepinephrine administration and the septic-shock mortality. In addition, the sample size was relatively small. Therefore, large multicenter prospective cohort studies are needed to validate whether early norepinephrine administration is associated with decreased mortality in patients with septic shock.

## Conclusions

This study indicates that early administration of norepinephrine for septic shock is associated with improved survival. Mortality increases when initial norepinephrine administration is delayed. Early norepinephrine initiation can increase MAP, shorten the duration of hypotension and, thereby, may improve vital organ perfusion and decrease serum lactate levels. These data suggest that more prompt and aggressive norepinephrine administration should be considered as part of initial resuscitation therapy for septic shock.

## Key messages

- Early administration of norepinephrine increases the survival rate of septic shock patients.
- Early norepinephrine initiation can increase MAP, shorten the duration of hypotension and, thereby, may improve vital organ perfusion and decrease serum lactate levels.
- The time to initial norepinephrine administration, the time to initial antimicrobial treatment, serum lactate level at the onset of septic shock, APACHE II score, intravenous fluid therapy within six hours and effective antimicrobial therapy are independently associated with hospital mortality.
- More prompt and aggressive norepinephrine administration should be considered as part of initial resuscitation for septic shock.

## Abbreviations

APACHE: Acute Physiology and Chronic Health Evaluation
CCM/ESICM/ACCP/ATS/SIS: The Society of Critical Care Medicine/the European Society of Intensive Care Medicine/the American College of Chest Physicians/ the American Thoracic Society/the Surgical Infection Society
CI: confidence interval
MAP: mean arterial pressure
NE: norepinephrine
OR: odds ratio
SSC: Surviving Sepsis Campaign
